# Rapid and automatic detection of micronuclei in binucleated lymphocytes image

**DOI:** 10.1038/s41598-022-07936-4

**Published:** 2022-03-10

**Authors:** Xiang Shen, Ying Chen, Chaowen Li, Fucheng Yang, Zhanbo Wen, Jinlin Zheng, Zhenggan Zhou

**Affiliations:** 1 School of Mechanical Engineering and Automation, Beihang University, Beijing, 100083 China; 2 Beijing Huironghe Technology Co., Ltd, Beijing, 101102 China

**Keywords:** Cytogenetics, Image processing, Machine learning, Software, Statistical methods

## Abstract

Cytokinesis block micronucleus (CBMN) assay is a widely used radiation biological dose estimation method. However, the subjectivity and the time-consuming nature of manual detection limits CBMN for rapid standard assay. The CBMN analysis is combined with a convolutional neural network to create a software for rapid standard automated detection of micronuclei in Giemsa stained binucleated lymphocytes images in this study. Cell acquisition, adhesive cell mass segmentation, cell type identification, and micronucleus counting are the four steps of the software's analysis workflow. Even when the cytoplasm is hazy, several micronuclei are joined to each other, or micronuclei are attached to the nucleus, this algorithm can swiftly and efficiently detect binucleated cells and micronuclei in a verification of 2000 images. In a test of 20 slides, the software reached a detection rate of 99.4% of manual detection in terms of binucleated cells, with a false positive rate of 14.7%. In terms of micronuclei detection, the software reached a detection rate of 115.1% of manual detection, with a 26.2% false positive rate. Each image analysis takes roughly 0.3 s, which is an order of magnitude faster than manual detection.

## Introduction

Once large-scale radiation accident occurs involving lot of people, it is critical to evaluate their exposure dose for further treatment^1^, so as to determine the priority for treatment and alleviate their unnecessary worries. Since ionizing radiation can cause chromosome damage, the biological dose can be estimated by cytogenetic methods. Dicentric chromosome detection^2^ is the most reliable cytogenetic dose estimating approach, but the analysis process is very time-consuming and requires excellent professional skills and knowledge. Therefore, when a large number of samples need to be evaluated, other dose estimating methods^3–7^ have aroused the interest of researchers. Among these other methods, CBMN analysis^8,9^ is the best alternative method.

CBMN analysis is a way of producing binucleate cells by adding a proper quantity of cytochalasin B to the cell culture process, causing the nucleus to divide normally while the cytoplasm does not. Since micronucleus (MN) is a fragment or an entire the chromosome, the binucleated cell's MN rate can be correlated with the exposure dose to estimate the unknown dose^10–13^, similar to the detection of chromosome aberrations. When the cell is irradiated, MN is in a hysteresis condition in the late stages of nuclear division. As a result, MN does not reside in the main nucleus of the cell, but rather forms a tiny circular entity and remains in the cytoplasm^14^. Compared with dicentric chromosome detection, CBMN analysis is easier and faster, making it is more appropriate for rapid biological dosimetry in a large number of persons. In addition to radiation dose estimation, CBMN analysis is also an effective detection method for studying the potential genotoxicity and cytotoxicity of chemicals^15,16^. Due to the subjectivity and long assay time of manual detection^17,18^, manual detection cannot satisfy the demands of rapid standard detection. To overcome these problems and produce superior statistical data and reproducibility, automated detection of MN has become a critical topic.

Currently, there are a variety of automated analysis methods for detecting binucleated cells (BNCs) and micronuclei (MNi). One of the most important distinctions between automated analysis algorithms is whether or not the studied object comprises cytoplasm. The first sort of algorithm only analyzes just the nucleus information and does not correlate the cytoplasmic information of related cells^19–29^, which is suitable for images captured by automated microscopes^19–23^ and imaging flow cytometers^24–29^. When employing imaging flow cytometers, only fluorescent dyes can be used to mark cell nuclei and micronucleus, and the detection method can be the conventional gating strategy^27^ or the deep learning CNN algorithm^28,29^. When using automatic microscopes, both fluorescent dye and Giemsa dye can be utilized for cell staining. Since Giemsa marks both the nucleus/MN and the cytoplasm, it will have an inescapable effect on the nucleus/MN recognition. Therefore, fluorescent staining is frequently favored when employing automatic microscopes^20,21^. This algorithm uses a threshold algorithm to determine the location of the cell nucleus, then the distance between each nucleus to determine the location and range of the cell, and finally the conventional gating strategy or a pre-trained convolutional neural network (CNN) model to determine the type of cell and the number of MNi.

The second kind of algorithm makes the entire cell as the detecting unit^30–37^. This algorithm is more in line with BNC detection criteria^38^ and is appropriate for images captured by automatic microscopes. When it comes to cell staining, the dye must not just stain the nucleus/MN, but the entire cell as well. The visualized nucleus and cytoplasm can be used to match the nucleus/MN with the corresponding cells to identify BNCs and MNi more accurately. This algorithm separates the image backdrop, cytoplasm, and nucleus/MN using different pixel thresholds, then employs a gating strategy to determine the cell's integrity and the number of nuclei/MNi^30,31^. Because the staining residue of Giemsa can cause blurred cytoplasmic background and produce some artifacts similar to MN, some researchers stain the nucleus and cytoplasm separately with two fluorescent dyes^32^ or use higher magnification imaging technologies to reduce the false positive incidence of MN^35,36^.

The current study has demonstrated that automatic detection of MN has engineering application value, but existing algorithms still have some drawbacks, such as low MN detection rates. In this study, a rapid standard automatic analysis software for Giemsa stained binuclear lymphocyte images based on automatic microscope imaging was designed using CNN. Low hardware costs and repeatable slide scanning are two advantages of automated microscopes. Giemsa dyes are economical, and the slides can be kept for years. Furthermore, the algorithm can distinguish BNCs and MNi even when the cytoplasm is hazy, and it can also detect monocytes (MOCs) and multinucleated cells (POCs).

## Materials and methods

### Software and hardware platform

**Figure 1:**
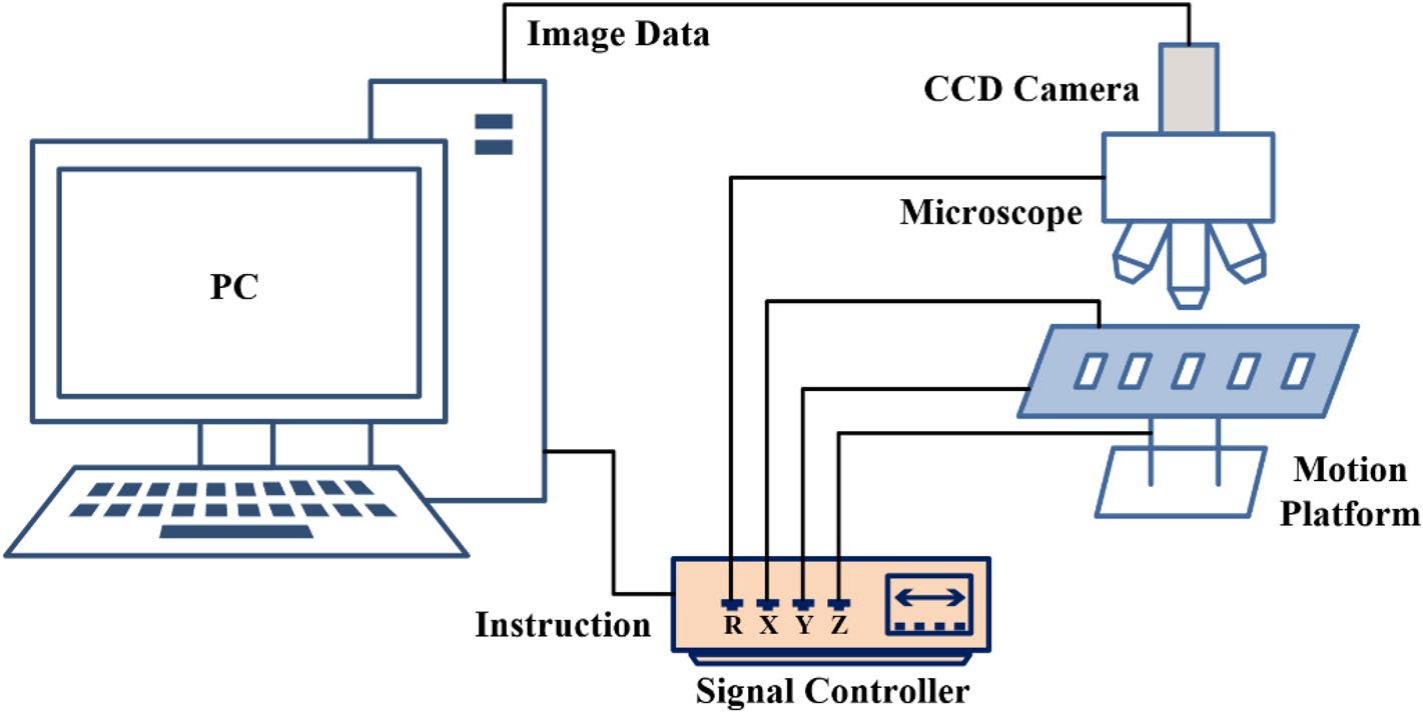
CBMN rapid automatic analysis system.

To implement rapid CBMN analysis, high-definition microscopic images of the cells must be obtained at the very beginning. The image acquisition system is made up of three parts (Fig. 1): (1) A Color microscope (OLYMPUS, Japan) with 10x, 20x, and 40× objective lenses. (2) A CCD camera (Lumenera, Canada), which is connected to the computer through the USB interface to allow for real-time display and picture taking. (3) A computer and a motion platform.

The computer sends commands to the motion console through Ethernet to move it along the predefined path. The microscope travels up and down to acquire photos that are then saved in the computer. 3200 pictures can be captured by scanning an entire standard-size glass slide using a 10× eyepiece and a 20× objective lens, depending on the camera magnification and the size of the normal glass slide.

On a desktop computer with a 6-core Intel Core i7-8700K 3.70 GHz CPU (32G RAM) and NVIDIA GeForce GTX 1080 Ti GPU (11G memory), software development and picture analysis were carried out. The software development tool uses Visual Studio 2013 and PyCharm 2018. Image capture, image processing, image analysis, and data storage management are among the four modules included in the software. Open CV and other third-party libraries are included in the software support library. C++ uses Python scripts to realize the categorization of distinct sorts of cells.

### Slide source

China Henan Institute of Occupational Disease Prevention and Control was entrusted for manufacturing 100 samples of CBMN. All donors are nonsmokers who have been maintained generally well at the time of blood collection, have no visible ailments such as the common cold, flu, or pneumonia, and have not been exposed to medical treatment in the previous 12 months. Informed consent was obtained from all subject and/or their legal guardian. A 4 Gy gamma irradiation was applied to all blood samples.

All experimental protocols of this research submitted in Scientific Reports were approved by the Ethic Committee of the China Henan Institute of Occupational Disease Prevention and Control and conformed to the Declaration of Helsinki, including:

- Collection of human blood.
- Cell culture and glass slide production.
- Collection of microscopic cell images.
- Automated detection and manual analysis of binuclear lymphocyte images.

The slide samples were prepared in compliance with the standards and criteria set out by the International Atomic Energy Agency^2^.

### Image acquisition

The sample was imaged using an OLYMPUS microscope with a 20 × objective. Each field of vision received one image, which had a size of 2048 × 2048 pixels and was saved in BMP format. There were 100 slides marked for model training and testing in total. 80 out of 100 slides from 16 donors (8 males, 8 females) were used for CNN model training, whereas the residual 20 slides from the other 4 donors (2 males and 2 females) were kept untouched until the final testing. The training-marked slides were scanned and photographed by our system.

## Design

There are four phases in the picture analysis process. First, extract the cells from the image background. Then, segment individual cells from the adhesive cell masses. Then, use the trained CNN model to detect and categorize individual cells. Finally, count the number of MNi of the BNC.

### Cell acquisition

**Figure 2:**
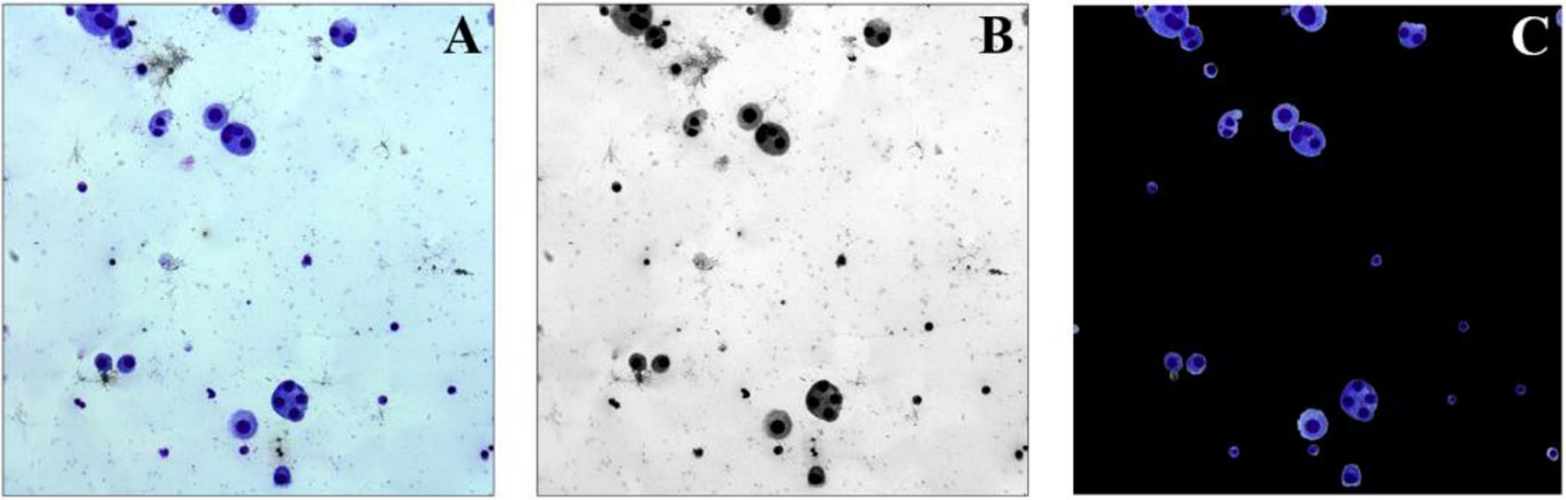
Image thresholding segmentation process. (**A**) Color original image. (**B**) Grayscale image of G channel conversion. (**C**) A composite result obtained by mask image and original color image.

Threshold segmentation is done on the acquired picture initially to generate a full cell image. The original color image (Fig. 2A) was transformed into grayscale images of R, G, and B channels according to the RGB channels before threshold segmentation. The image of the sample will seem dark blue or reddish purple in human vision since it has been stained with Giemsa. As a result, the cytoplasm/nucleus pixel intensity in the G channel grayscale picture (Fig. 2B) is the highest among the four images of R, G, B grayscale images and conventional grayscale photos. The picture is then thresholded using the iterative thresholding approach for the G-channel gray image. Each image (2048 × 2048 pixels) takes roughly 0.2 s to process using the iterative threshold classification algorithm. The mask image is eroded and extended after thresholding to eliminate certain minor noise spots and contaminants. Finally, the mask image is combined with the original color image, as seen in Fig. 2C.

### Classification of individual cells and adhesive cell masses

Since the cell distribution on the glass slide is not uniform, sticky cell masses will appear in the photos produced in III.A. To improve BNC detection rates, the adhesive cell mass must be segmented into individual cells. Before segmentation, all of the acquired cells must be classified into individual cells and adherent cell clusters.

There are two aspects of the classification method that are evaluated: 1. Each cell should have a complete cytoplasm and be nearly round; 2. The area value of the cell should be within a reasonable range. Based on these issues, cell area, cell extension rate, and cell defect rate are used as judgment variables.

(1) The cell area threshold is used to filter out tiny impurities and big adherent cell clusters. These impurities or cell clusters will significantly increase the algorithm's analysis time.

The area threshold's lower limit is about the minimum area value of a cell (1000 pixels, which is comparable to around 100 μm^2^), while the upper limit is approximately the area value of 5 cells (8000 pixels) (when the number of adhering cell clusters is more than 5, the adhesion situation is complicated and the cell detection rate of the watershed algorithm is low.). These variables can remain constant after the microscope magnification and camera specifications have been determined. A series of thresholds have been tested as shown in Supplementary Table S2.

(2) The calculation formula of the elongation rate parameter is:

1 $$\mathrm{min}\left(\mathrm{H},\mathrm{ W}\right)/\mathrm{max}(\mathrm{H},\mathrm{ W})$$

where H is the width of the cell's smallest circumscribed rectangle and W is the length. The elongation rate is a measurement of how near an object's form is to a circle. The elongation value of a circle is 1; for things that are not as round, the elongation value is less than 1, and as the form of the item approaches non-circular, the elongation value approaches 0.

(3) The formula for calculating the defect rate is:

2 $$ \left( {{\text{A}} - {\text{B}}} \right)/{\text{B}} $$

where A is the area of the cell's smallest circumscribed circle, and B is the cell's total area. The elongation rate indicates how close the object's real area is to the circular area. The defect rate for a circle is 0; for cell objects with incomplete cytoplasm, the defect rate is higher.

The elongation rate and defect rate thresholds are used to separate the images into two types: individual cells and adherent cell clusters (the impurities will be further identified in the CNN model). A large number of images will be assessed as adhesion cell clusters if the parameter setting is too stringent (for example, the elongation rate is 0.8–1.0). The watershed technique will be used to segregate these images, slowing down the algorithm's analysis pace and lowering the BNC detection rate (some intact cells are over-segmented). If the parameters are set too loosely (for example, 0.2–1.0), many adherent cell clusters that need to be separated will be evaluated as individual cells, reducing the number of cells found by the algorithm. A variety of thresholds were tested in the process of defining parameter thresholds, as shown in Supplementary Table S1.

The two judgment parameters are combined in a "parallel" way, which means that when an item's elongation rate parameter and defect rate parameter are both within the allowed range, the object is regarded as an individual cell. When the values in the group 4 of Table S2 (the Elongation rate is set to 0.5–1.0, the Defect rate is set to 0–0.5, and the area threshold is set to 1000–8000) are used, it is more consistent with the definition of intact cells^38^, and an ideal balance between the algorithm's cell detection rate and the analysis speed can be achieved.

### Adhesive cell masses segmentation

The adhesion cell masses were segmented using the watershed algorithm^39^ in this step. To get the distance gradient map, first grayscale the image of the sticky cell mass, and then continually corrode the grayscale image. Finally, the edge lines of each cell in the image of the sticky cell mass are obtained using the watershed method on the distance grayscale image.

**Figure 3:**
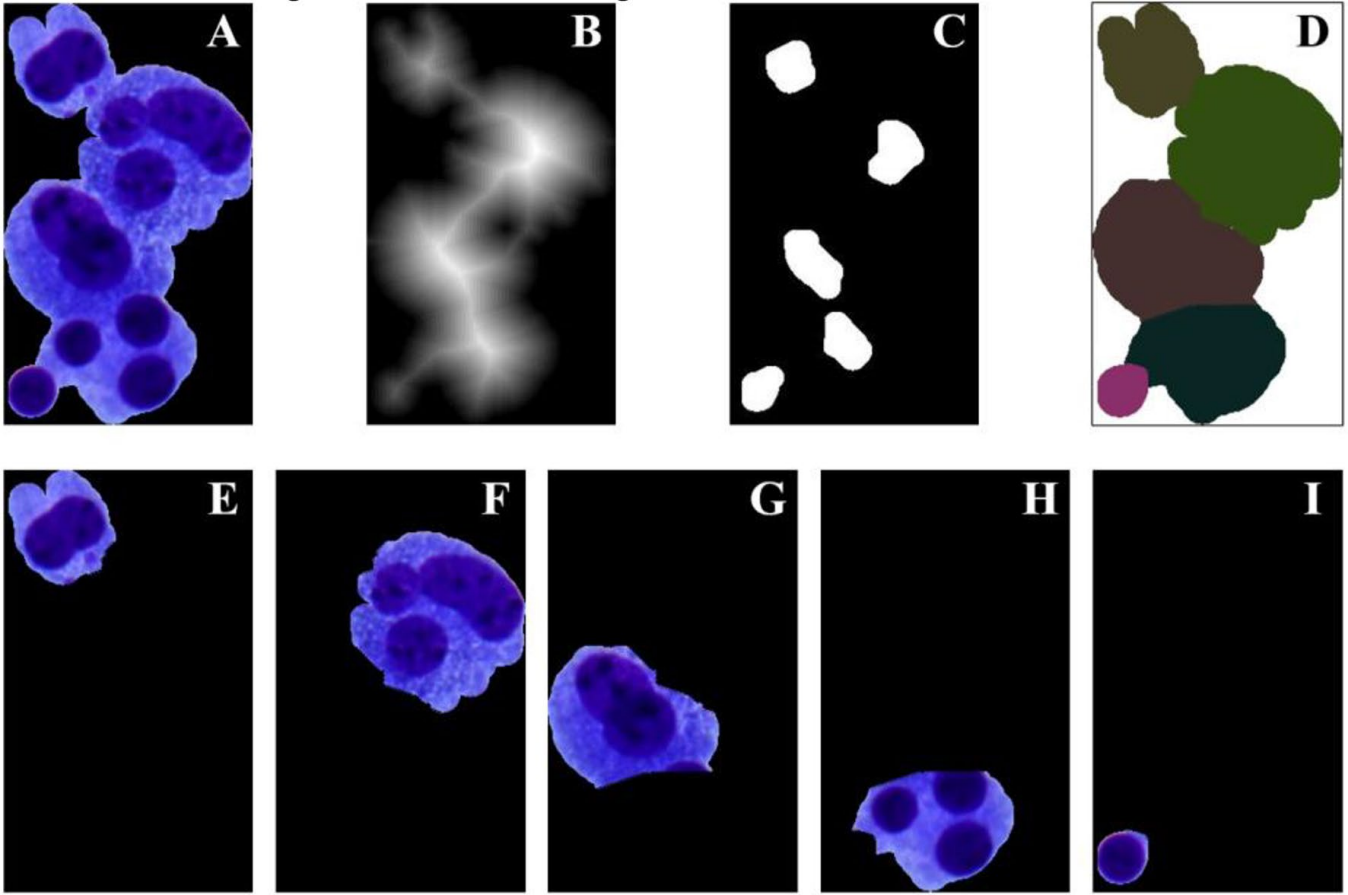
Segmentation results of adhesion cell clusters. (**A**) Original image of adhesive cell mass. (**B**) Distance gradient map. (**C**) Seed points map. (**D**) The image after segmentation. (**E**–**I**) Individual cell images after segmentation.

The use of traditional watershed algorithm will result in excessive segmentation since the form and size of the cells varies, and certain contaminants and cells stick together. As a result, the seed point area threshold judgment was added to the algorithm. To avoid excessive segmentation, the number of pixel points of the seed point was used as the criteria, and spurious seed points with too tiny area values were rejected. The lowest seed point threshold was set at 100 pixels in this algorithm. Figure 3 shows the adhesion cell cluster segmentation findings.

### Cell type recognition based on CNN model

CNN is made up of image input layer, convolutional layer, maximum pool, average pool, comprehensive connection layer, and other components as a deep learning algorithm. It employs mapping to take the original pixels of the input picture as input, extracts features in a hierarchical way, and then classifies the features using a fully connected layer. The number of layers can be raised or lowered depending on the size of the input picture, and it is not required to use all of the layers in the network. CNN has the benefit of not requiring any artificial feature extraction using other approaches.

**Figure 4:**
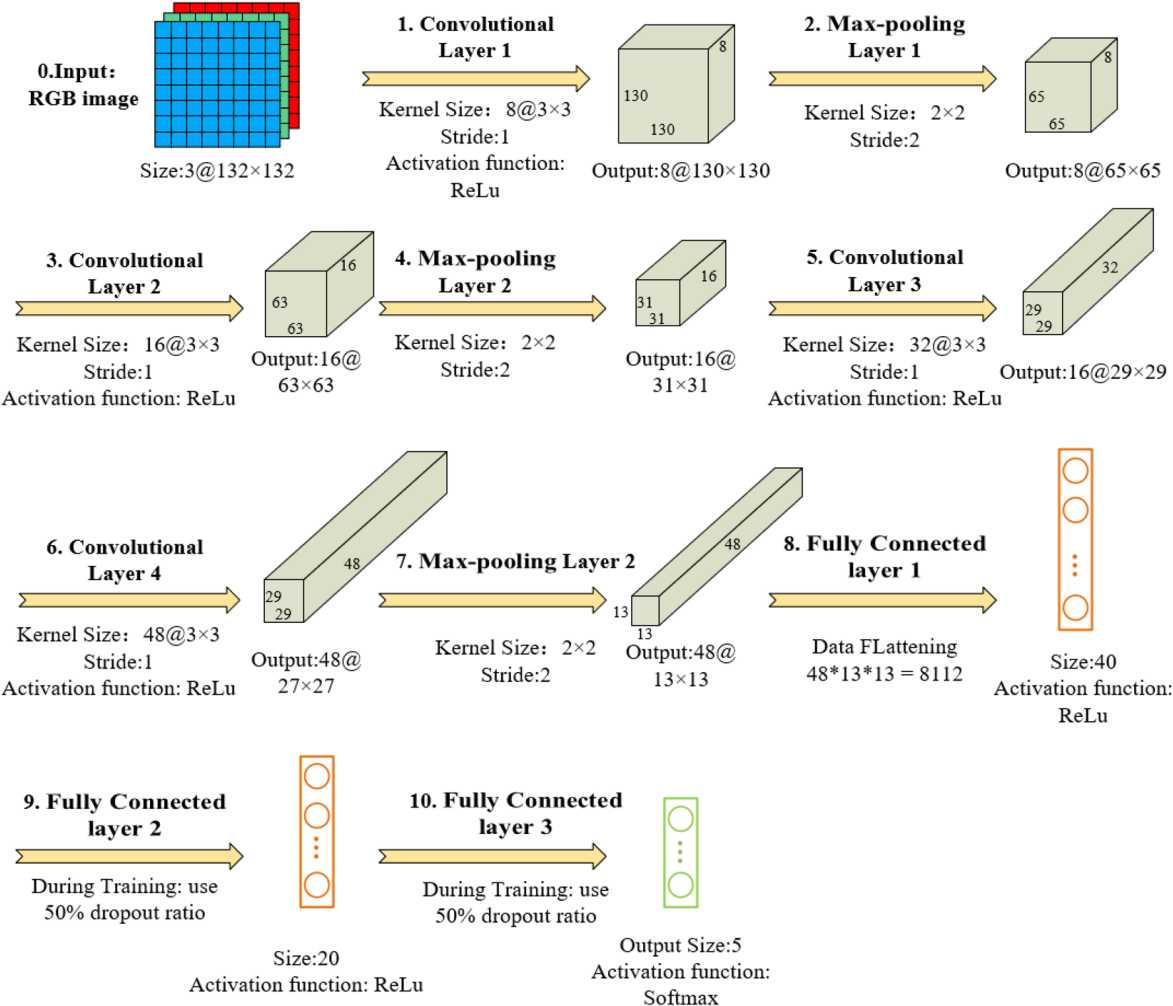
Overview of the proposed CNN architecture.

A CNN model for image categorization of micronucleus cells is developed, and the suggested CNN architecture is shown in Fig. 4. There are seven layers in this CNN model (4 convolutional layers, 3 fully connected layers). The first convolutional layer (Conv1) uses 8 kernels with a size of 3 × 3 to filter a 3 × 132 × 132 input image. Conv2 has 16 filters with a size of 3 × 3, and Conv 3 and Conv 4 have 32 and 48 filters with a size of 3 × 3, respectively. The first fully connected layer (FC1) has 40 neurons, and each neuron uses a 50% loss rate during training. FC2 has 20 neurons, and each neuron also uses a 50% loss rate during training. After Conv1, Conv2, and Conv4, the largest pooling layer with a 2 × 2 kernel size is used. The rectified linear unit (ReLU) activation function is used after each convolution and fully connected layer. In the end, the softmax function is used to generate probability distributions on the five class labels.

On the images of 80 slides used for training, about 280,000 images of individual cells were produced after conducting cell extraction and segmentation of adhesion cell clusters. Five experienced analysts carried out the categorization according to defined guidelines^38^. Cell images were divided into five categories: 0. BNC without MN, 1. Binucleated cell with Micronuclei (BNCMN), 2. MOC, 3. POC with three nuclei and above, 4. Any other cell or substance that does not belong to the first four types. 24,000 BNC images without MN, 16,000 BNCMN images, 45,000 MOC images, 35,000 POC images, and 160,000 other types of images were obtained in the end. The amount of BNC and BNCMN photos is only 14% of the total number of images, and the number of different types of images is not evenly distributed. To increase the model's capacity to detect BNC/BNCMN images, the BNC/BNCMN images are oversampled by 2 times and 4 times, respectively. In addition, to balance the corresponding category photos in the training set, data augmentation methods were utilized to produce new images. Horizontal flip, random addition of noise or blocks with occlusion effects on the original picture, main component assay (PCA) dimensionality reduction processes are some of the techniques employed. Two dropout layers were inserted between the FC1 and FC2 layers (dropout = 0.5) and between the FC2 and FC3 layers (dropout = 0.5) of the model to reduce over-fitting caused by picture over-sampling. According to a 7:2:1 ratio, each type of picture was randomly sorted into a CNN training set, validation set, and test set. The training and validation sets are involved in the CNN model training process, but the test set is not involved in the model training and just acts as a preliminary internal test.

Since the area values of individual cell pictures varies, all photos must be scaled to a 132 × 132 sized image without stretching before training (132 × 132 is close to the average area of BNC images). Then use TensorFlow's Keras function package to implement the CNN model. The learning rate was initialized to 0.001 and reduced by a factor of 10 after half of the iterations. The momentum term was set to 0.9, and the weight decays was set to 0.0001. All parameter choices, with the exception of the given configuration, are standard setups. The network was trained on the NVIDIA GeForce GTX 1080 Ti GPU for 60 iterations in 40 h. The model's convergence was good during the training phase, and there was no noticeable over-fitting problem. In the end, the model's total recognition accuracy was 85%, while the loss rate was around 0.4.

**Figure 5:**
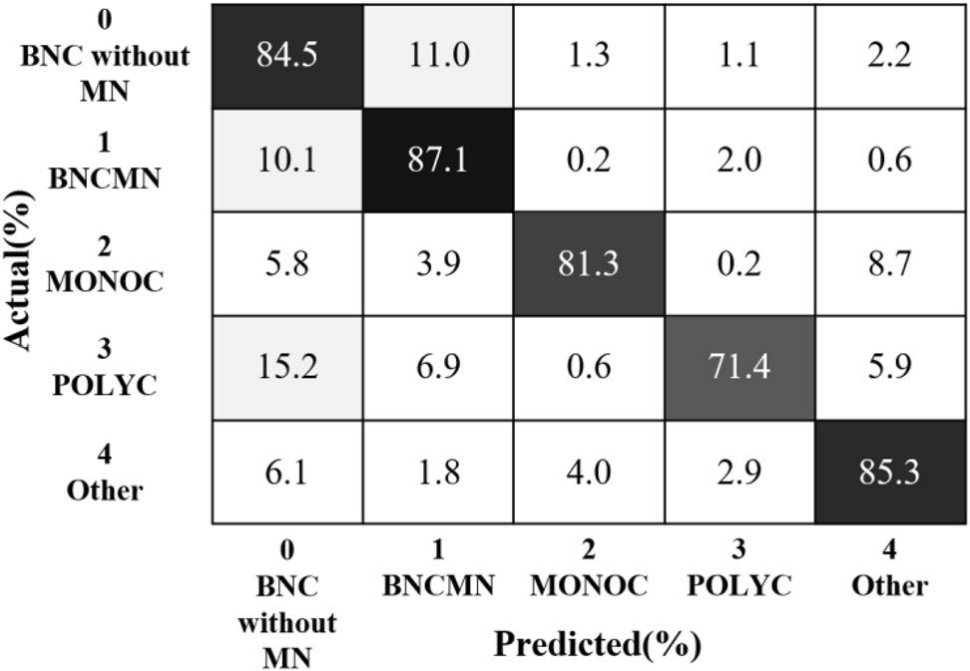
Confusion matrix of recognition results.

The trained model was internally tested using the images of the test set. BNC images without MN had a recognition accuracy of 84.5%, and BNCMN images had a recognition accuracy of 87.1%. Figure 5 depicts the test results for 5 different categories.

### Micronucleus count

The CNN model in III.D can only identify cell types, but it didn't determine how many MNi are in a BNC with several MNi. Therefore, the number of MNi must be determined at this step of the work. The nucleus and MNi were extracted from the cytoplasm first, and then the numerous MNi that were joined attached and the MNi that were attached to the nucleus were segmented. Finally, the nuclei and MNi were sorted by area value to count the number of MNi.

**Figure 6:**
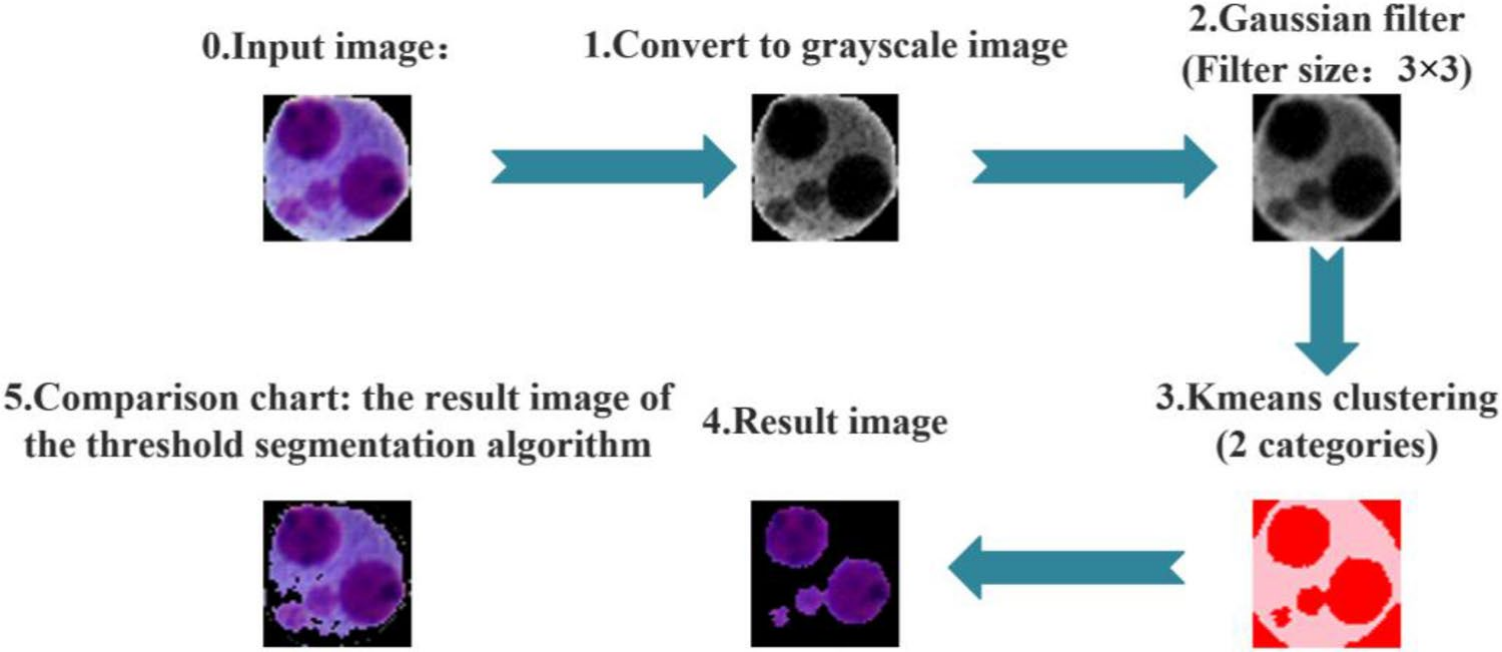
Flow chart of extracting nuclei and MNi.

The iterative threshold technique cannot properly extract nuclei and MNi from these images since there are many images of cells with hazy cytoplasm in the gathered images. As a result, a *k*-means clustering-based threshold segmentation technique was developed and Fig. 6 depicts the flowchart. Pure black (image background), light purple (cytoplasm), and dark purple (nucleus/ MN) are the three hues in the cell picture. The black background has a gray value of 0, the nucleus/MN has a gray value near to 0, and the cytoplasm has a gray value greater than 0. *K* was set to 2 to guarantee that the clustering method can successfully differentiate the cytoplasm from the nucleus/MN since the gray value difference between the black background and the nucleus/MN is lower than the difference between the nucleus/MN and the cytoplasm.

**Figure 7:**
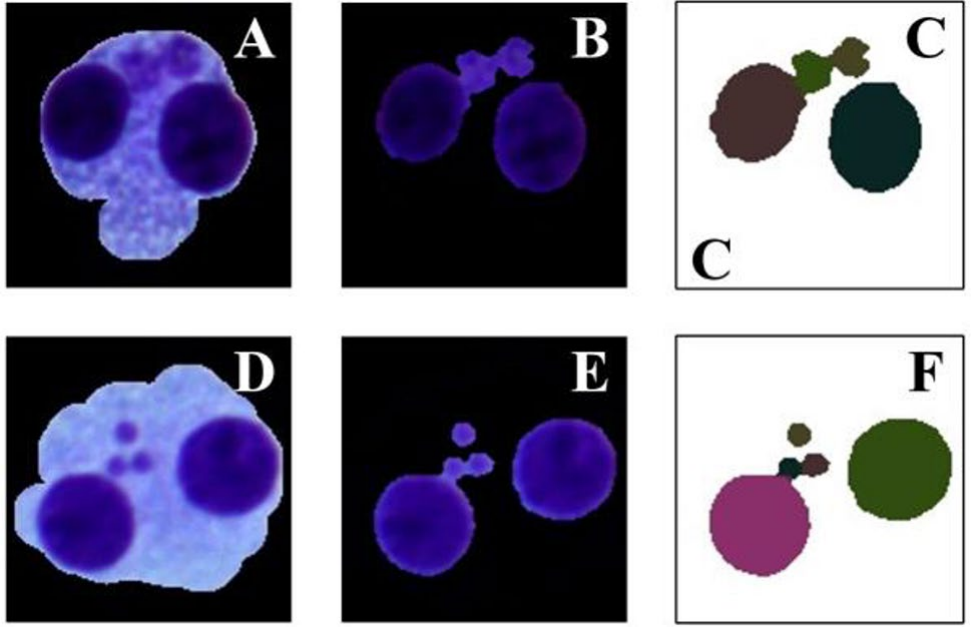
The resulting graph after using *k*-means clustering threshold algorithm and modified watershed segmentation algorithm. (**A**,**D**) Original image. (**B**,**E**) The image after extracting cell nucleus and MN. (**C**,**F**) The image after segmentation.

After extracting the nuclei and MNi, import them into the watershed algorithm. The corrosion parameters of the watershed algorithm used here are different from the watershed algorithm in III.C. Because the goal of III.C is to segment the adhesive cell masses, a higher corrosion parameter (value: 7) is required. The major purpose in this stage is to separate the adhesive MN masses into independent MNi, not to divide the adhering nucleus masses into independent nuclei (since the number of nuclei has already been determined). To intensify the segmentation effect, the watershed algorithm utilizes a smaller corrosion parameter (value: 3) in this stage. Figure 7 depicts the segmentation effect. The area value and coordinate information of each nucleus/MN can be acquired when the segmentation is complete.

When the area information of the cell nucleus and MNi have been obtained, they are ordered numerically.

Following that, the potential MNi are screened out based on the assumption that the maximum permitted MN area is 20% of the nuclear area value (if there are several nuclei, the average value of multiple nuclei is chosen). Finally, compute the number of MNi based on the premise that the smallest allowed MN area is 20 pixels (to prevent some noise points from being misjudged as MN).

## Results

### Random image algorithm test

Twenty slides from 4 separate donors (2 men and two females, A-D) were scanned and photographed, and the photos were then utilized to test the system. These 20 slides' images, as noted in II.C, did not take part in the CNN model's training. Following the completion of the image collection, 100 images were chosen at random from each slide's picture collection, 500 images for each donor, for a total of 2000 images.

The proofreaders also performed artificial visual scoring on 2000 original images according to the standard method^38^. The image analysis data obtained is shown in Table 1. Five experienced inspectors detected 1949 BNCs without MN, 1029 BNCsMN, and 1359 MNi, as indicated in Table 1. In all, the manual detection required around 7.5 h of proofreading time. These images are then automatically analyzed by the software, and the results are displayed in Table 2.

**Table 1:** The number of Binucleated Cells (BNCs), Binucleated Cells with Micronucleus (BNCsMN), Micronuclei (MNi) and the number of Micronuclei per Binucleated Cell (MNi/BNCs) by manual scoring.

Donors	Slide numbers	BNCs	BNCsMN	MNi	MNi/BNCs
A	A 1	106	36	39	0.368
	A 2	116	40	50	0.431
	A 3	107	37	43	0.402
	A 4	118	40	47	0.398
	A 5	119	42	52	0.437
B	B 1	136	47	62	0.456
	B 2	132	46	57	0.432
	B 3	139	48	67	0.482
	B 4	138	48	64	0.464
	B 5	140	49	62	0.443
C	C 1	164	57	76	0.463
	C 2	171	59	63	0.368
	C 3	161	55	79	0.491
	C 4	163	56	83	0.509
	C 5	165	57	80	0.485
D	D 1	177	60	88	0.497
	D 2	179	59	75	0.419
	D 3	183	63	90	0.492
	D 4	180	66	89	0.494
	D 5	184	64	93	0.505
Total number		2978	1029	1359

**Table 2:** The number of BNCs, BNCsMN, MNi and MNi/BNCs through automatic scoring.

Donors	Slide numbers	BNCs	BNCsMN	MNi	MNi/BNCs
A	A 1	127	39	63	0.496
	A 2	130	40	65	0.500
	A 3	122	37	60	0.492
	A 4	125	39	56	0.448
	A 5	124	38	53	0.427
B	B 1	131	41	73	0.557
	B 2	125	38	74	0.592
	B 3	129	40	67	0.519
	B 4	136	42	70	0.515
	B 5	130	40	60	0.462
C	C 1	150	46	90	0.600
	C 2	156	48	74	0.474
	C 3	145	45	82	0.579
	C 4	156	49	84	0.538
	C 5	148	45	76	0.514
D	D 1	183	57	101	0.552
	D 2	184	63	93	0.505
	D 3	183	56	103	0.563
	D 4	190	59	113	0.595
	D 5	187	58	107	0.572
Total number		2961	920	1564	

The automatic image analysis took roughly 620 s in total (about 0.3 s per image), which is 44 times faster than manual detection. 2041 BNCs without MN, 920 BNCsMN, and 1564 MNi were recognized by the software.

Following the automatic analysis of these images, the same proofreaders utilize the system's gallery to visually proofread the BNC images acquired from the automated analysis and count the result's error scores.

Table 3 shows the proofreaders' right diagnosis. Table 4 shows the relationship between automatically scored data and proper diagnosis by observers.

**Table 3:** The detection result of automatic counting.

	BNCs	BNCsMN	MNi
Automated scoring	2961	920	1564
Manual diagnosis	2525	816	1154
Manual scoring	2978	1029	1359
Total number of cells (5 categories)		23,941	
BNCs yield relative to manual scoring		99.4%	
BNCsMN yield relative to manual scoring		89.4%	
MNi yield relative to manual scoring		115.1%

**Table 4:** Relation between the automatically scored data and correct diagnosis by observers.

	BNCs	Non-BNCs	MNi	Non-MNi
Automated scoring	TP = 2525	FP = 436	TP = 1154	FP = 410
	FN = 453	TN = 20,527	FN = 205	TN = 4763
	BNCs ACC	96.3%	MNi ACC	90.6%
	BNCs TPR	84.8%	MNi TPR	84.9%
	BNCs TNR	97.9%	MNi TNR	92.1%
	BNCs PPV	85.3%	MNi PPV	73.8%
	BNCs NPV	97.8%	MNi NPV	95.9%

For model estimation, the quality parameters (Accuracy, Sensitivity, Specificity, Positive Predictive Value, Negative Predictive Value) were utilized. These parameters can be calculated via

3 $$ {\text{Accuracy }}\left( {{\text{ACC}}} \right) = \left( {{\text{TP}} + {\text{TN}}} \right)/\left( {{\text{TP}} + {\text{FP}} + {\text{TN}} + {\text{FN}}} \right) $$

4 $$ {\text{Sensitivity }}({\text{true positive rate}},{\text{ TPR}}) = {\text{TP}}/\left( {{\text{TP}} + {\text{FN}}} \right) $$

5 $$ {\text{Specificity }}({\text{true negative rate}},{\text{ TNR}}) = {\text{TN}}/\left( {{\text{TN}} + {\text{FP}}} \right) $$

6 $$ {\text{Positive Predictive Value }}({\text{PPV}}) = {\text{TP}}/\left( {{\text{TP}} + {\text{FP}}} \right) $$

7 $$ {\text{Negative Predictive Value }}({\text{NPV}}) = {\text{TN}}/\left( {{\text{TN}} + {\text{FN}}} \right) $$

TP: Ture Positive; FP: False Positive; FN: False Negative; TN: Ture Negative.

Table 5 shows the right diagnosis made by proofreaders. Table 6 shows the relationship between the gating strategy's automated scoring data and proper diagnosis by observers.

**Table 5:** The detection result of gating strategy.

	BNCs	BNCsMN	MNi
Scoring by gating strategy	1727	478	712
Manual diagnosis	1587	450	662
Manual scoring	2978	1029	1359
Total number of cells (5 categories)		23,941	
BNCs yield relative to manual scoring		58.0%	
BNCsMN yield relative to manual scoring		46.5%	
MNi yield relative to manual scoring		52.4%

**Table 6:** Relation between the detection data by gating strategy and correct diagnosis by observers.

	BNCs	Non-BNCs	MNi	Non-MNi
Automated scoring	TP = 1587	FP = 140	TP = 662	FP = 50
	FN = 1391	TN = 20,823	FN = 697	TN = 5123
	BNCs ACC	93.6%	MNi ACC	88.6%
	BNCs TPR	53.3%	MNi TPR	48.7%
	BNCs TNR	99.3%	MNi TNR	99.0%
	BNCs PPV	91.9%	MNi PPV	93.0%
	BNCs NPV	93.7%	MNi NPV	88.0%

It has been found that utilizing an automated analysis approach based on the CNN model, the identification rate of BNC is significantly higher than using a conventional image recognition algorithm (99.4% vs 58.0%). Since most BNCs with two sticky cores are discarded, conventional image recognition algorithms achieve a high PPV (91.9% vs 85.3%) by systematically eliminating potential targets. As compared to the conventional gating strategy algorithm, the algorithm based on CNN tends to behave more adventurous (high TPR and low TNR).

There are no adjustments for age, sample storage duration, or the treated or untreated instances of patients, since the difference between cases and controls is not the major purpose of this study. And it has no effect on the direct comparison of visual and automated counting.

### Assay of test results by SPSS

For studying the difference between the results of automatic analysis and manual detection, SPSS (Statistical Product and Service Solutions, version 19.0) was used to perform statistical analysis on the MN rate (MNi/BNCs) data in Tables 1 and 2. The 20 MN rates in Table 1 were regarded as the "Manual" group, and the 20 MN rates in Table 2 were regarded as the "Automatic" group. Since the amount of data is not large, and the two sets of data do not obey the normal distribution, the Mann–Whitney U was first used to test whether there is a statistical difference between the MN rate data of manual visual inspection and automatic analysis.

In the Mann–Whitney U test results, the average rank of the manual detection data is 13.30, and the average rank of the automatic analysis data is 27.70. The exact *P* value is < 0.001 (The specific data can be viewed in Supplementary Fig. S1). As a result, the statistical significant difference between manual detection and automatic analysis was determined. According to calculations, it can be found that the mean and median values of the data of the automatic analysis group are higher than the manual detection group, indicating that the value of the automatic grouping is generally higher than the manual detection group.

**Figure 8:**
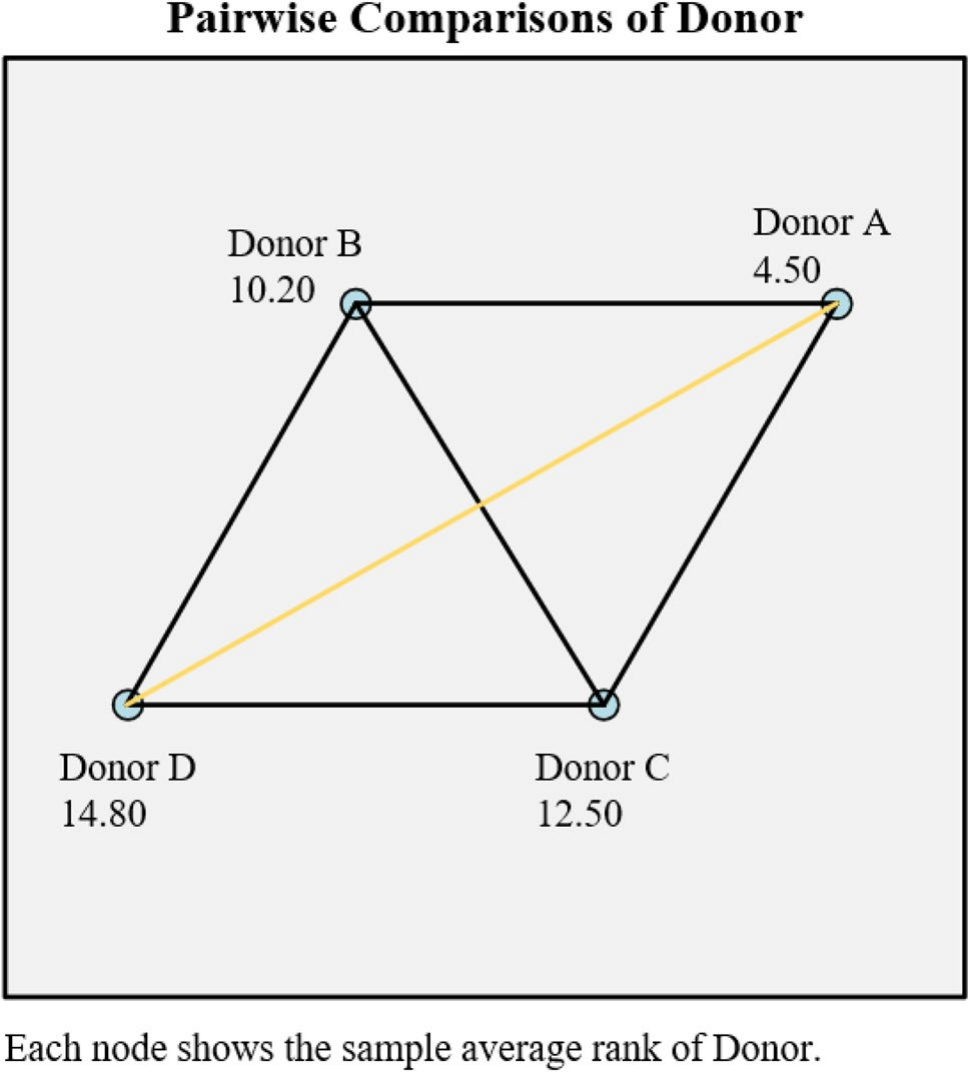
The pairwise comparison result of manually detected MN rate data.

Then, the 20 MN rates in Table 1 were divided into four groups from A to D according to their respective donors (the number of samples in each group is 5). Since the amount of data is not large, and each group of data does not follow a normal distribution, the Kruskal–Wallis H test is performed. On the one hand, the Kruskal–Wallis H test can detect whether there are statistically significant differences between the 4 groups of data. On the other hand, two conclusions can be drawn using the Kruskal–Wallis H test on the MN rate data of manual detection and automatic analysis. The consistency between the two conclusions may then be shown. If both inspection conclusions are the same, it proves that the automatic analysis has the detection ability roughly equivalent to the manual detection. The test result data of the MN rate of manual detection is shown in Fig. 8.

It can be found that the distribution of the manually detected MN rate data is not the same, and the difference is statistically significant (*P* = 0.039 < 0.05, in Supplementary Fig. S2). The average rank of Donor A group data is 4.50, the average rank of Donor B group data is 10.20, the average rank of Donor C group data is 12.50, and the average rank of Donor D group data is 14.80.

**Figure 9:**
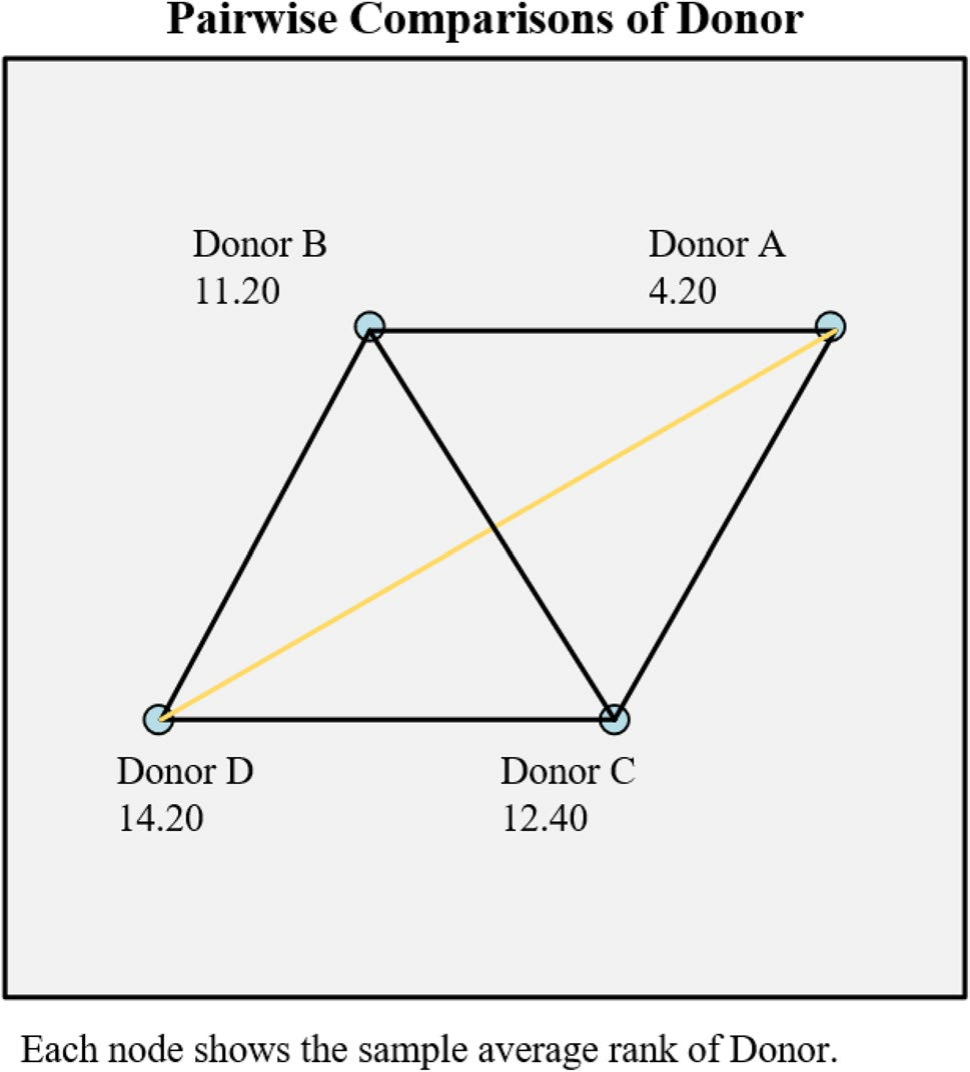
The pairwise comparison result of automatically analyzed MN rate data.

After the significance level was adjusted by Bonferroni method, the data of each group were compared in pairs, and it was found that the data distribution of Donor A group and Donor D group (*P* = 0.035 after adjustment, *P* < 0.05) was statistically different, while the difference between the other groups was not statistically significant (the comparative data of other groups can be viewed in Supplementary Fig. S3). Similarly, the 20 MN rate data in Table 2 are also divided into four groups from A to D (n = 5) according to their respective donors. The test results are shown in Fig. 9.

It can be found that the distribution of the data of each group of the automatic analysis result is not completely the same, and the difference is statistically significant (*P* = 0.042 < 0.05, in Supplementary Fig. S4). The average rank of Donor A group data is 4. 20, the average rank of Donor B group data is 11.20, the average rank of Donor C group data is 12.40, and the average rank of Donor D group data is 14.20. After the significance level was adjusted by Bonferroni method, the data of each group were compared in pairs, and it was found that the data distribution of Donor A group and Donor D group (*P* = 0.045 after adjustment, *P* < 0.05) was statistically different, while the difference between the other groups was not statistically significant (The comparative data of other groups can be viewed in Supplementary Fig. S5).

In Fig. 8, the value next to the dot represents the average rank of the group. The connecting line represents the result of the pairwise comparison, the black connecting line indicates the difference between the two groups is not statistically significant, and the orange connecting line indicates the statistical significance of the difference between the two groups.

The conclusions of the Kruskal–Wallis H test for manually detected and automatically analyzed data are same. Both conclusions concluded that the data distributions of Donor A and Donor D are statistically significantly different. As a result, the automatic CBMN analysis algorithm has a detection capacity that is roughly equivalent to manual detection.

## Discussion

The purpose of this research is to design a system that can automatically analyze Giemsa stained CBMN images and analyze the images while capturing. This method, unlike conventional gating strategy analysis algorithms, preserves cells to a higher extent, allowing it to detect more BNCs/MNi and be more adventurous. Conventional image recognition steps like manual feature extraction, dimensionality reduction, and feature ranking are also not needed. Because CNN can intelligently alter network settings to get the greatest classification performance and extract components autonomously by producing feature maps after each convolutional layer, allowing it to find the best performance elements on its own. A *K*-means clustering-based cell nucleus/MN extraction approach is developed to overcome the problem of CNN being unable to count MNi of BNCs with multiple MNi. The algorithm can also handle the adherence of numerous MNi to one another as well as the adhesion of MNi to the nucleus. The automatic CBMN analysis method detected BNCs and BNCsMN at 99.4% and 89.4% of manual detection, respectively, with BNCs false positive rates of 14.7%.

The most of pseudo-BNCs come from cells with overlapping complicated nuclei. When confronted with such images, even seasoned observers have difficulty determining whether the nuclei overlap or connect in space. MN had a detection rate of 115.1% as compared to manual detection, and a PPV of 73.8%. The most common source of false positives in MN is Giemsa dye, which causes a fuzzy cytoplasmic background and artifacts similar to MNi. These artifacts are also very difficult for manual detection. Some MNi with lighter hues, on the other hand, are overlooked by the algorithm since they are difficult to distinguish using the *k*-means clustering threshold approach. The identical Kruskal–Wallis H test result of manual detection and automatic analysis MN rate data demonstrates that the automatic CBMN analysis algorithm has detection capacity that is roughly equivalent to manual detection. The algorithm's detection accuracy and rate can be enhanced further by increasing the number of CNN training samples, eventually reaching a level that is totally comparable to that of experienced human observers.

Some algorithms^19–29^ in prior similar work only analyzed nuclear data. To establish the cell border, whether using the gating strategy^19–27^ or the CNN algorithm^28,29^, it is important to examine the relative position and size of the cell nucleus. When this type of algorithm is employed, it is typically required to prioritize the use of fluorescent stains^20,21^ in the slide production process and to limit cell density as much as possible to avoid the circumstance where some nuclei are accidentally assigned to the same cell^22^. The conventional gating strategy algorithm involves first searching the nucleus, then judging the two neighboring nuclei as a BNC, defining the circular region around the two nuclei of the BNC as the cell area, and then searching for MNi in this area. The gating strategy's determination parameters are based on morphological characteristics such as area^22^, circle^26^, overlap of two nuclei^25^, and so on, and this approach detects MN at a rate of 20–35%^20,24,25^ of manual counting. Other algorithms^30–37^ in prior similar work used the entire cell as the detection unit. This type of algorithm first identifies the cytoplasm of the cell to determine whether it is intact^30,31,37^, then detects the number of nuclei in the cell, and lastly counts the number of MNi. The MN detection rate analyzed by this sort of algorithm is about 65% of the manual count^30,35^. Our algorithm flow is similar to that of previous similar work that uses the entire cell as the detection unit. The detection rate of our system is higher than that of the prior system^20,30^ because it combines adhesion cell cluster segmentation and a binucleated cell identification strategy based on CNN, both of which were not included in previous similar work.

In terms of slide applicability and system clinical application, this system may obtain satisfactory identification results for slides created using conventional procedures without requiring high staining effect^33^ or cell density criteria^31^. The system can also detect BNCs and MNi with blurred cytoplasmic without utilizing a high microscope magnification, indicating that it has both great detection and speedy analysis capabilities. Unlike conventional image analysis algorithms, this software uses a CNN model for cell recognition. As a result, no tedious manual feature selection or threshold parameter adjustment is required, and all that is required is to manually give enough training data for CNN.

According to statistics, the system takes roughly 620s to analyze 2000 pictures, which is 44 times faster than manual detection. With enough BNCs in slide, the typical analysis time for biological dose estimation is just 7–10 min if completed according to the criteria of studying a minimum of 1000 BNCs. This unquestionably enhances the speed of CBMN analysis when compared to manual visual evaluation. Faster image processing rates can be achieved by installing the program on high-end workstations and further improving system performance.

Not only BNCs, but also a large number of MOCs and POCs are produced in the CBMN process. Another benefit of this system is that it can distinguish between cells with differing nucleus numbers. The cell proliferation index^40^, the ratio of trinuclear cells to tetranuclear cells^41^, and other information may be calculated using MOC and POC data, which can aid in the estimation of high radiation doses.

## Conclusion

This study describes a method for analyzing Giemsa stained CBMN microscopic images quickly and automatically, with a high detection rate and processing speed. This system is simple to use and does not need specifical control over cell density and staining intensity. The BNC and BNCMN detection rates of this system were 99.4% and 89.4%, respectively, of manual detection, while the analysis speed was 40–50 times faster than manual detection. Even when the cells are heavily colored, the method can distinguish BNCs from MNi and MOCs from POCs. As the number of training samples grows, CNN can consistently enhance MN's accuracy and detection rate.

In the future, system testing and analysis will be carried out on samples irradiated with various dosages in order to continuously enhance the system's performance. The software's detection capabilities will also be improved, allowing for the detection of MOCs containing MNi, POCs containing MNi, nucleoplasmic bridges, necrotic cells, and apoptotic cells.

## Supplementary Information

Supplementary Information.
