# Supplementary material for: Rapid and automatic detection of micronuclei in binucleated lymphocytes image
Source: Sci Rep. 2022 Mar 10;12:3913. doi: 10.1038/s41598-022-07936-4 (PMC8913785; doi:10.1038/s41598-022-07936-4)
Supplement: Supplementary file 1 — Supplementary Information. [file 41598_2022_7936_MOESM1_ESM.docx]

# Supplementary information


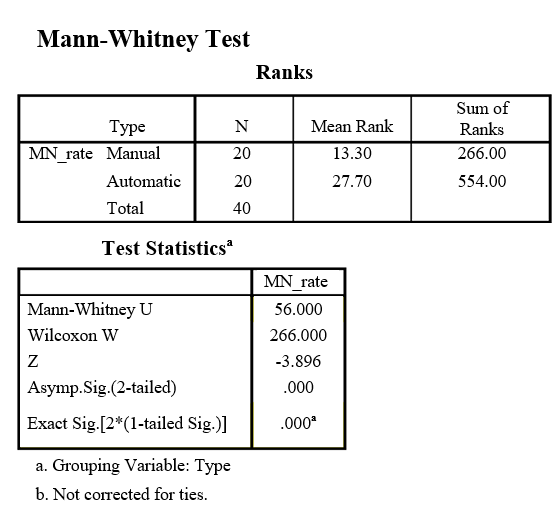


**Figure S1.** Mann-Whitney U test results


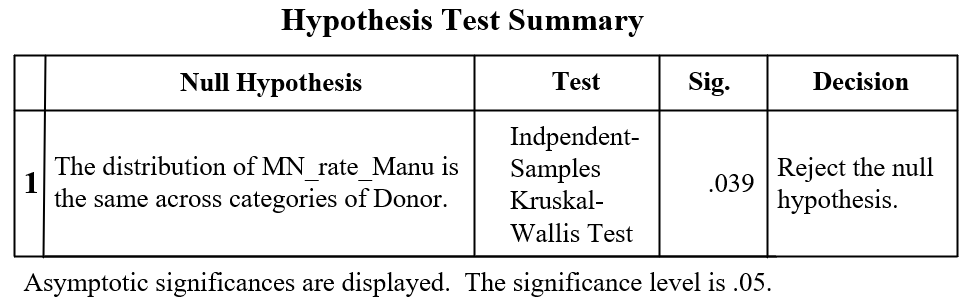


**Figure S2.** Kruskal-Wallis H hypothesis test results of manually detected MN rate data


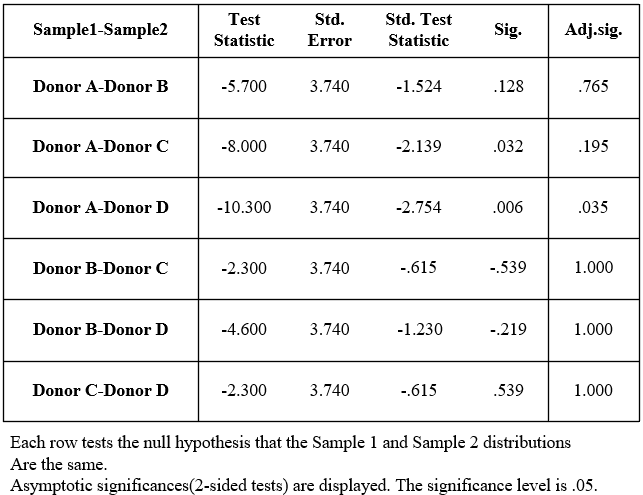


**Figure S3.** Results of Pairwise Comparisons of MN rate of manual detection


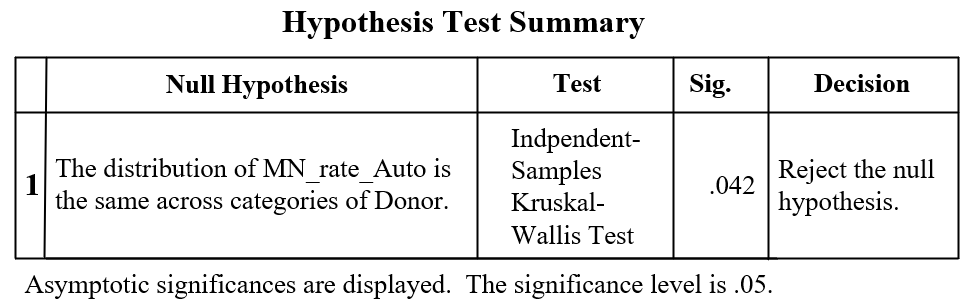


**Figure S4.** Kruskal-Wallis H hypothesis test results of automatically analyzed MN rate data


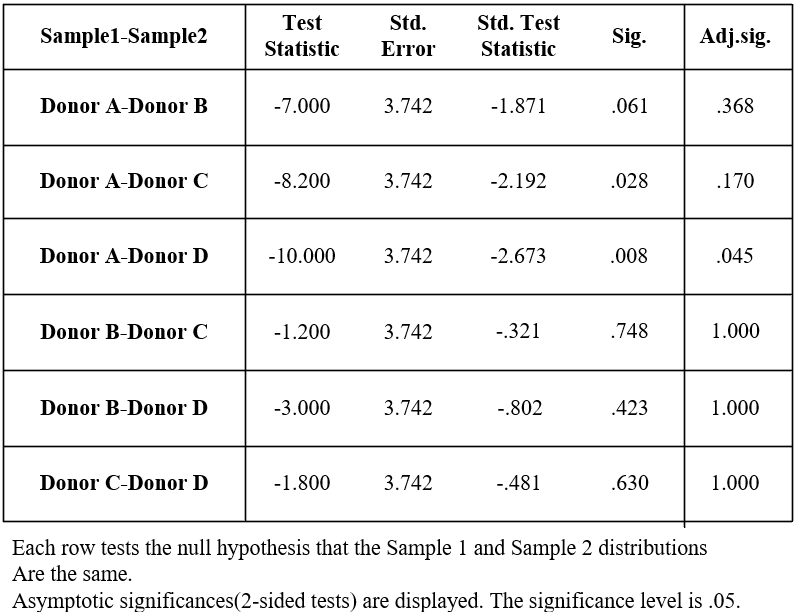


**Figure S5.** Results of Pairwise Comparisons of MN rate of automatic analysis

**TABLE S1.** The relationship between the detection parameters and the number of cells in 2000 pictures (The area threshold is set to 1000-6000)

| Parts | ER | DR | BNC number | Cell number | BNC rate |
| --- | --- | --- | --- | --- | --- |
| A | 0.25-1 | 0-0.25 | 711 | 20351 | 3.5% |
|  |  | 0-0.5 | 728 | 21862 | 3.3% |
|  |  | 0-0.75 | 732 | 23173 | 3.2% |
| B | 0.5-1 | 0-0.25 | 735 | 21945 | 3.3% |
|  |  | 0-0.5 | 741 | 23616 | 3.1% |
|  |  | 0-0.75 | 716 | 23441 | 3.1% |
| C | 0.75-1 | 0-0.25 | 730 | 21847 | 3.3% |
|  |  | 0-0.5 | 715 | 23376 | 3.1% |
|  |  | 0-0.75 | 735 | 23588 | 3.1% |
| D | 0.5-1 | 0-0.45 | 730 | 22739 | 3.2% |
|  | 0.5-1 | 0-0.55 | 739 | 23581 | 3.1% |
|  | 0.45-1 | 0-0.5 | 734 | 23661 | 3.1% |
|  | 0.55-1 | 0-0.5 | 732 | 23590 | 3.1% |

ER: Elongation rate, DR: Defect rate, BNC: Binucleated Cells

**TABLE S2.** The relationship between the area threshold and the number of cells in 2000 pictures (The Elongation rate is set to 0.5-1.0, the Defect rate is set to 0-0.5)

| Group number | Lower limit | Upper limit | Time | BNC number | BNC rate |
| --- | --- | --- | --- | --- | --- |
| 1 | 500 | 6000 | 347 s | 743 | 2.5% |
| 2 | 1000 | 4500 | 224 s | 735 | 3.1% |
| 3 | 1000 | 6000 | 237 s | 741 | 3.1% |
| 4 | 1000 | 8000 | 247 s | 750 | 3.1% |
| 5 | 1000 | 10000 | 273 s | 750 | 3.1% |
| 6 | 1000 | 12000 | 298 s | 752 | 3.1% |

Lower/Upper limit: the lower/upper limit of area threshold
